# Association between personality traits, eating behaviors, and the genetic polymorphisms *FTO*-rs9939609 and *MAO-A* 30 bp u-VNTR with obesity in Mexican Mayan children

**DOI:** 10.3389/fgene.2024.1421870

**Published:** 2024-07-26

**Authors:** Luis Alberto Vázquez-Pérez, Mónica Hattori-Hara, Gloria Arankowsky-Sandoval, Gerardo Pérez-Mendoza, Rodrigo Rubi-Castellanos, Jorge Aarón Rangel-Méndez, Doris Pinto-Escalante, Thelma Canto-Cetina, Lizbeth González-Herrera

**Affiliations:** ^1^ Centro de Investigaciones Regionales “Dr. Hideyo Noguchi”, Universidad Autonoma de Yucatan, Mérida, Mexico; ^2^ Secretaría de Educación del Gobierno del Esatdo de Yucatán (SEGEY), Mérida, Mexico

**Keywords:** childhood obesity, eating behavior, personality traits, *FTO*-rs9939609, *MAO-A* 30 pb u-VNTR, Mayan children

## Abstract

**Introduction:**

Genetic variants that control dopamine have been associated with obesity in children through loss of control of satiety and impulses, the manifestation of addictive eating behaviors, and specific personality traits. The variants include *FTO*-rs9939609 and the *MAO-A* 30 pb u-VNTR low-transcription alleles (LTA).

**Objective:**

To evaluate the genetic association of *FTO*-rs9939609 and the *MAO-A* LTA, along with personality traits and eating behavior with obesity in Mayan children from Mexico.

**Methods:**

We cross-sectionally evaluated 186 children (70 with obesity and 116 with normal weight) 6–12 years old from Yucatan, Mexico. Nutritional status was defined with body mass index (BMI) percentiles. Personality traits were evaluated with the Conners and TMCQ tests; eating behavior was evaluated with the CEBQ test. Genotyping with real-time PCR and TaqMan probes was used for *FTO*-rs9939609, whereas PCR amplification was used for *MAO-A* u-VNTR.

**Results:**

High-intensity pleasure (*p* = 0.013) and moderate appetite (*p* = 0.032) differed according to nutritional status. Heterozygous *FTO*-rs9939609 T/A children showed higher mean scores of low-intensity pleasure (*p* = 0.002) and moderate appetite (*p* = 0.027) than homozygous T/T. Hemizygous boys having *MAO-A* LTA showed significantly higher mean scores of anxiety (*p* = 0.001) and impulsivity (*p* = 0.008). In multivariate models, only LTA alleles of *MAO-A* explained obesity in boys (OR = 4.44; 95% CI = 1.18–16.63).

**Conclusion:**

In the present study, *MAO-A* u-VNTR alleles were associated with obesity in multivariate models only in boys. These alleles might also have a role in personality traits such as anxiety and impulsivity, which secondly contribute to developing obesity in Mayan boys.

## 1 Introduction

Overweight and obesity are defined as an abnormal and excessive accumulation of fat that might lead to health impairment. Fat accumulation in the early stages of development is related to later non-communicable diseases onset, such as diabetes, arterial hypertension, cardiovascular disease, and cancer (Umer et al., 2017; Hidayat et al., 2018). Thus, investigating the origin of childhood obesity would prevent morbidity in adolescents, adults, and older adults. Accordingly, some research has focused on evaluating the genetic and psychological aspects of childhood obesity, although from a non-integrative perspective.

It has been demonstrated that the psychological characteristics of children lead to behavior-related body fat excess (Flores-Dorantes et al., 2020). Such factors are configured by genetic-environment interactions that have shown paradoxical results: obese children have common personality traits that are not distinctive of a unified profile or a specific mental disease when compared with their normal weight counterparts (Shaker et al., 2014). However, obese children exhibit more significant anxiety and depressive symptoms arising from low self-esteem, distorted body image, and being victims of bullying (Lindberg et al., 2020). Taking together, anxiety, impulsivity, and the search for stimulation, reward, gratification, or novelty become the personality traits related to obesity.

On the other hand, eating behavior refers to the set of actions that underlies the relationship of the human being with food. This factor is mainly learned from parent behavior, although these patterns are associated with impulsivity, anxiety, and the search for stimulation and reward (Kininmonth et al., 2021). Moreover, the extensive study of genetic impairment has led to identifying genes related to impulsivity and searching for reward or gratification, particularly those associated with the serotoninergic and dopaminergic systems. Given the primary role of the dopaminergic rewarding process in appetite modulation, it has been shown that genetic variants of dopamine-related enzymes, namely, the fat mass and obesity-related (*FTO*) gene and the monoamine oxidase A (*MAO-A*) gene, might be associated with palatable food and, therefore, with obesity physiopathology (Fuemmeler et al., 2008; Qi et al., 2014; Dias et al., 2016; Krishnan et al., 2017). It has also been suggested that genetic variants associated with obesity partly exert their influence on BMI and waist circumference through undesirable eating behaviors such as disinhibition and susceptibility to hunger (Jacob et al., 2018).

The *FTO* gene is located on the locus 16q12.2 and consists of nine exons and eight introns. It is expressed in the hypothalamus, hypophysis and suprarenal glands, muscle, and adipose tissue. The latter is relevant since the *FTO* has been implied in fat mass regulation through the lipolysis mechanism (Jacobsson et al., 2008) and the adipocyte early differentiation process (Claussnitzer et al., 2015). The most studied variant from *FTO* is the SNP rs9939609 (g.87653T>A), in which carriers of the risk allele A (homozygous or heterozygous) have increased odds for obesity and higher calorie, fat, and carbohydrate intake (Daya et al., 2019; Mehrdad et al., 2020). Moreover, overweight children carrying the A allele have shown higher scores on the Food Responsiveness, Emotional Overeating, Enjoyment of Food, and Food Choice subscales and lower scores on the Satiety-Responsiveness and Slowness in Eating subscales. Obese children have shown higher scores on the Cognitive Restrained subscale and lower Food choice (Obregón Rivas et al., 2018). Minor allele frequency of *FTO-*rs9939609 has been reported in 16.8% of individuals from Yucatan, Mexico (Hernandez-Escalante et al., 2014).

Otherwise, monoaminoxidase (MAO) is a mitochondrial isoenzyme that catalyzes amine and neurotransmitter oxidation such as serotonin, norepinephrine, dopamine, phenylethylamine, and others (Bortolato et al., 2008). Humans produce two forms, MAO-A (mainly in catecholaminergic brain neurons) and MAO-B (mainly in brain serotoninergic and histaminergic neurons), that possess selective affinity by neurotransmitters. For example, MAO-A exhibits a higher affinity for serotonin, norepinephrine, and dopamine, whereas MAO-B is selective for phenylethylamine (Bortolato et al., 2008). The *MAO-A* consists of 16 exons in chromosome X (Xp11.23). It contains a functional 30 bp upstream variable number of tandem repeats (30 bp u-VNTR) located in the promoter region and consists of 2, 3, 3.5, 4, or 5 repeated copies, with the 3- (3R) and 4-repeat (4R) alleles as the most common. Given the central role of MAO-A in modulating neurotransmitters, it might be hypothesized that an impact on eating behavior, such as impulsivity, anxiety, and neuroticism, might result in obesity. In this respect, the lower activity 3R allele has been reported to be associated with obesity in Caucasian populations (Fuemmeler et al., 2008; Wallmeier et al., 2013; Dias et al., 2016); conversely, the 4R allele has been associated with higher consumption of lipid and sugar dense foods in Brazilian boys (Galvão et al., 2012).

In Mexico, 75.2% of adults (≥20 years) are overweight (39.1%) or obese (36.1%). Strikingly, 37.3% of children (5–11 years) exhibit the former (19.2%) or latter (18.1%) condition (Shamah-Levy et al., 2023). Of note is the case of Yucatan, Mexico, where 30% of children (5–11 years) are overweight or obese. This situation ranks this Mexican State among the 10 States with higher rates of these conditions (Shamah-Levy et al., 2023). Data from a national survey showed an elevated prevalence of consumption of “not recommended” food in children, mainly sugar-sweetened beverages (92.9%) (Shamah-Levy et al., 2023). Moreover, it has been reported that 87% of Mexican children and adolescents follow a dietary pattern high in fat and sugar (Galvan-Portillo et al., 2018). Even the *FTO-*rs9939609 has been found to be associated with obesity in Mayan girls (González-Herrera et al., 2019b). Thus, it might be hypothesized that the excessive consumption of such foods might be triggered by genetic impairment of the serotoninergic and dopaminergic pathways related to personality traits and eating behavior. Hence, the present study aimed to evaluate the genetic association between *FTO-*rs9939609 and *MAO-A* 30 bp u-VNTR variants, along with personality traits and eating behavior with obesity in Mayan children from Mexico.

## 2 Materials and methods

### 2.1 Subjects

School-aged children with Mayan ethnicity (6–12 years old) attending public elementary schools at Southeast, Mexico (Yucatan), were enrolled from January and June 2017. A total of 186 children participated, 52.7% were girls and 47.3% were boys. We included 116 children with normal weight and 70 children with obesity according to the World Health Organization (WHO) growth charts for children aged 5–19 years (De Onis et al., 2007).

All children were measured for weight with a digital scale (Seca, Hamburg, Germany) and height with a portable stadiometer (Seca 225, Hamburg, Germany) in a standing position without shoes. BMI-for-age was obtained from the WHO growth reference data tables for 5–19 years, and then it was z-scored for standard deviation (SD) calculation. Normal-weight infants were those with a BMI-for-age between 15th and 84th percentiles (BMIpc) or between −1 and < +1 SD, whereas infants with obesity were those with a BMI-for-age >97th percentile or ≥ +2 SD (De Onis et al., 2007).

Children with chronic diseases, monogenic obesity, as well as children with a BMI-for-age between +1 SD and +2 SD (overweight) or less than −1 SD (underweight) were excluded. Selected children were born in Yucatan, having familial ancestors back for at least the third generation born in Yucatan. Children with Mayan ethnicity were selected using anthropological and demographic parameters, such as language, birthplace, surnames, and genealogy, to match ethnically all children. We previously reported the genetic ancestry of the population from the Yucatan Peninsula, where Mayan is the main Amerindian genetic background (González-Herrera et al., 2019a; Lara-Riegos et al., 2020).

This study was conducted according to the Declaration of Helsinki and its later amendments. It was approved by the Institutional Review Board of Bioethics of the Centro de Investigaciones Regionales “Dr. Hideyo Noguchi,” Universidad Autónoma de Yucatán, Mexico (CEI/03/2015). Patients’ personal information was kept strictly confidential, and only the principal investigator could access it; parents of selected children signed informed consent. However, they had to provide assent for the venipuncture procedure.

### 2.2 Personality traits and eating behavior evaluation

Personality traits and eating behavior in children were evaluated from parents’ perspective. Questionnaires were answered by 86.6% of mothers and 13.4% of fathers. Impulsivity and anxiety were evaluated with a rigorous selection of items from the Conners test (Keith Conners et al., 1998), whereas low (LIP) and high (HIP) intensity pleasure were measured with some items from the Temperament in Middle Childhood Questionnaire (TMCQ) (Rothbart et al., 2001).

Eating behavior was evaluated through the Child Eating Behavior Questionnaire (CEBQ), translated and validated by our research group for its application in the Mexican population (Vázquez-Pérez et al., 2020). The adapted version included 34 items for six dimensions: 1) food responsiveness (FR) and emotional overeating (EOE) were condensed into the new “voracity and emotional intake (VEI)” category; 2) satiety responsiveness (SR) and slowness in eating (SE) were condensed into the new “moderate appetite (MA)”; 3) enjoyment of food (EF); 4) desire to drink (DD); 5) emotional undereating (EUE); and 6) food fussiness (FF).

### 2.3 Genotyping

Genomic DNA was extracted from peripheral whole blood using a conventional in-house method. The *FTO-*rs9939609 was genotyped using the TaqMan^®^ Allelic Discrimination Assay (Applied Biosystems, Foster City, CA, United States) C_30090620_10 in a real-time PCR StepOne device (Applied Biosystems) following supplier specifications. All PCR reactions were done in 48-well plates with a final volume of 10 μL. The allelic discrimination was done with the StepOne v.2.1 software.

The *MAO-A* 30 bp u-VNTR was evaluated using the Lung et al. method (Lung et al., 2011). Briefly, the PCR reaction was performed, according to manufacturer instructions, in a final volume of 25 μL with the Dream Taq Green PCR Master Mix (Thermo Scientific) and the forward (5′-ACA​GCC​TCG​CCG​TGG​AGA​AG-3′) and reverse (5′-GAA​CGG​ACG​CTC​CAT​TCG​GA-3′) primers. The PCR products were visualized in 10% acrylamide gels stained with silver nitrate. With this method, the sizes for the 2R, 3R, 4R, and 5R allelic variants were 320 bp, 350 bp, 380 bp, and 410 bp, respectively.

*FTO-*rs9939609 was genotyped in 186 DNA samples, whereas *MAO-A* 30 bp u-VNTR was genotyped in 134 samples due to DNA quality.

### 2.4 Statistical analyses

Genotypic and allelic frequencies of *FTO-*rs9939609 and *MAO-A* u-VNTR polymorphisms were stratified by sex and BMI status and described with frequencies and percentages. Otherwise, the *MAO-A* 30 bp u-VNTR was stratified according to its associated transcriptional activity: the hemizygous boys with 3R and 4R alleles were included in the low (LTA) or high (HTA) transcriptional activity group, respectively. Girls with the 3R/3R genotype were considered the LTA, whereas the 3R/4R and 4R/4R genotypes were HTA. The Hardy-Weinberg equilibrium (HWE) was analyzed with a χ^2^ test at *p* > 0.05.

Personality traits and eating behavior scores were obtained from parent recalls and are presented as mean ± standard deviation (SD). These mean ± SD scores were compared between BMI status (obese vs. normal weight) and *MAO-A* 30 bp u-VNTR with Student’s t-test at *p* < 0.05; one-way ANOVA with Tukey’s *post-hoc* test at *p* < 0.05 was employed for comparisons between *FTO-*rs9939609 genotypes. Frequency comparisons were done with a χ^2^ test at *p* < 0.05.

Finally, the combined effect of sex, age, genetic variants, personality traits, and eating behavior on BMI status was evaluated in a multivariate logistic regression model in which odds ratio (OR) and 95% confidence intervals (95% CI) at *p* < 0.05 were calculated.

## 3 Results

Anthropometric description of the studied group according to BMI status is shown on Table 1. The mean ± SD age and BMIpc were 8.69 ± 1.4 years and 72.59 ± 27.83, respectively. BMIpc and weight significantly differed between obese and normal-weight infants (*p* < 0.001). Accordingly, 58.57% of boys and 41.43% of girls were obese. There was a significant difference for the frequency of obesity between boys and girls (*p =* 0.017), being the girls with a higher frequency of normal weight [*N =* 69 (59.48%)].

**TABLE 1:** Anthropometric variables according to BMI status. Mean ± SD.

Variables	Total (*N* = 186)	Normal weight (*N* = 116)	Obesity (*N* = 70)	*p-*value
Sex Boys, *N* (%) Girls, *N* (%)	88 (47.31) 98 (52.69)	47 (40.52) 69 (59.48)	41 (58.57) 29 (41.43)	0.017^a^
Age (years)	8.69 ± 1.94	8.63 ± 1.9	8.81 ± 2	0.545^b^
Heigh (cm)	131.63 ± 16.59	130.67 ± 12.13	133.39 ± 22.67	0.356^b^
Weight (kg)	35.16 ± 12.79	29.31 ± 7.24	45.9 ± 13.83	<0.0001^b^
BMI percentile	72.59 ± 27.83	56.26 ± 24.39	97.08 ± 6.28	<0.0001^b^

BMI: body mass index; SD: standard deviation.

^a^ χ test.

^b^ Student’s t-test.

### 3.1 Genotyping of *FTO-*rs9939609 and *MAO-A* u-VNTR according to sex and BMI status

The genotypic and allelic frequencies of *FTO-*rs9939609 according to sex are shown in Supplementary Table S1. The T/T genotype and the T allele were prevalent among the general sample; the polymorphism was not in HWE (*p* = 0.04), particularly in girls (*p* = 0.001). It has been reported that when the HWE deviations cannot be attributed to genotyping errors, selection, or non-random mating and may be caused by an unknown factor, the χ^2^ test for trend (Armitage trend test) should be investigated to reduce the chance of false-positive associations. Hence, after conducting the Armitage test, no deviations from the HWE were observed (*p* ≥ 0.93) (Alnafjan et al., 2022). Table 2 depicts the distribution of genotypic and allelic frequencies of *FTO*-rs9939609 according to sex and BMI status. There were no significant differences in genotypes/alleles according to BMI in girls, boys, or the total sample (*p ≥* 0.11).

**TABLE 2:** *FTO*-rs9939609 and *MAO-A* u-VNTR according to sex and BMI status.

Sex	BMI	*FTO*-rs9939609^a^					Sex	BMI	*MAO-A* u-VNTR^a^				
		T/T	T/A	A/A	T	A			3R/3R	3R/4R	4R/4R	3R	4R
Boys	Normal weight (*N =* 47, 2*N* = 94)	34 (72)	11 (24)	2 (4)	79 (84)	15 (16)	Boys	Normal weight (*N =* 33)^b^	N/A	N/A	N/A	14 (42)	19 (58)
	Obese (*N =* 41, 2*N* = 82)	34 (83)	7 (17)	0 (0)	75 (91)	7 (9)		Obese (*N =* 30)^b^	N/A	N/A	N/A	24 (80)	6 (20)
	*p*-value^c^	0.11			0.14			*p*-value^c^	N/A			**0.002**	
Girls	Normal weight (*N =* 69, 2*N* = 138)	58 (84)	7 (10)	4 (6)	123 (89)	15 (11)	Girls	Normal weight (*N =* 50, 2*N* = 100)	8 (16)	20 (40)	22 (44)	36 (36)	64 (64)
	Obese *N* (%) (*N =* 29, 2*N* = 58)	22 (76)	7 (24)	0 (0)	51 (88)	7 (12)		Obese (*N =* 21, 2*N* = 42)	5 (24)	10 (48)	6 (28)	20 (47.62)	22 (52.38)
	*p*-value^c^	0.12			0.81			*p*-value^c^	0.21			0.19	
Total	Normal weight *N* (%) (*N =* 116, 2*N* = 232)	92 (79.31)	18 (15.52)	6 (5.17)	202 (87.1)	30 (12.93)	Total	Normal weight (*N =* 83, 2*N* = 133)^b^	N/A	N/A	N/A	50 (38)	83 (62)
	Obese *N* (%) (*N =* 70, 2*N* = 140)	56 (80)	14 (20)	0 (0)	126 (90)	14 (10)		Obese (*N= 51*, 2*N* = 72)^b^	N/A	N/A	N/A	44 (61)	28 (39)
	*p*-value^c^	0.13			0.39			*p*-value^c^	N/A			**0.001**	

BMI, Body mass index. Significant *p*-values are in bold.

^a^ Data is presents as *N* (%).

^b^ Boys are hemizygous for *MAO-A* u-VNTR.

^c^ χ test.

Regarding the *MAO-A* u-VNTR, the most frequent genotype was the heterozygous 3R/4R (42.25%), and was in HWE (*p* = 0.33) (Supplementary Table S1). After stratifying by BMI status (Table 2), hemizygous boys with the 3R allele, which conferred the LTA, showed the highest prevalence of obesity (*p =* 0.002). These results were consistent in the total sample (*p* = 0.001) but not in girls (*p* ≥ 0.19). In terms of odds, boys carrying the 3R allele were 5.43 times more likely to be obese than those with the 4R allele (OR: 5.43, 95%CI: 1.75–16.81).

### 3.2 Personality traits and eating behavior according to BMI status

We did not find statistically significant differences in mean scores of personality traits and eating behavior according to BMI status, except for items of HIP and MA (Table 3). Children with normal weight obtained significantly higher mean scores than children with obesity for HIP (*p* = 0.013) and MA (*p* = 0.032), suggesting an eating behavior compatible with adequate satiety in non-obese children.

**TABLE 3:** Personality traits and eating behavior scores according to BMI status.

Psychological profile	BMI status		*p-*value^a^
	Normal weight	Obesity	
	N = 116	N = 70	
Personality traits, mean ± SD			
Anxiety	15.62 ± 4.20	15.89 ± 4.68	0.692
Impulsivity	17.61 ± 5.14	18.19 ± 5.59	0.481
High intensity pleasure	14.01 ± 6.14	11.61 ± 6.40	**0.013**
Low intensity pleasure	15.55 ± 4.10	15.60 ± 4.50	0.939
Eating behavior, mean ± SD			
Voracity and emotional intake	12.84 ± 7.92	15.10 ± 8.83	0.081
Moderate appetite	12.71 ± 5.94	10.94 ± 5.10	**0.032**
Enjoyment of food	12.48 ± 3.92	13.60 ± 3.80	0.056
Desire to drink	5.33 ± 3.14	5.23 ± 3.70	0.850
Emotional undereating	4.06 ± 2.95	3.64 ± 2.69	0.321
Food fussiness	7.48 ± 3.53	7.63 ± 3.72	0.786

^a^ Student’s t-test. Significant *p*-values are in bold.

### 3.3 Personality traits and eating behavior according to *FTO-*rs9939609 and *MAO-A* u-VNTR genotyping

Personality traits and eating behavior according to *FTO*-rs9939609 genotyping are shown in Supplementary Table S2. We found that children with heterozygous T/A genotype showed significantly higher mean scores in LIP (*p* = 0.002) and moderate appetite (*p* = 0.027) than homozygous T/T children. Homozygous A/A *FTO*-rs9939609 children showed the highest or the lowest score for all studied personality traits and eating behaviors, except EF; however, no significant difference was found compared with T/T wildtype *FTO* children (*p* > 0.05), which may be due to the low frequency of homozygous A/A in the studied population.

Data regarding *MAO-A* 30 bp u-VNTR and psychological features are shown in Supplementary Table S3. For the total population, only impulsivity was significantly higher in children with LTA alleles compared to the HTA (*p* = 0.027), suggesting that children carrying LTA alleles might develop a more impulsive personality. After stratifying by sex, girls did not exhibit any significant difference in mean scores of any personality trait nor any eating behavior after comparing HTA vs. LTA alleles (*p* > 0.05); the same was seen for girls with obesity (Supplementary Table S4) or normal weight (Supplementary Table S5). Otherwise, boys with LTA alleles exhibited significantly higher mean scores of anxiety (*p* = 0.001) and impulsivity (*p* = 0.008) than boys with HTA alleles, suggesting that LTA alleles might affect these personality traits only in boys (Supplementary Table S3). After stratifying by BMI status, only boys with obesity having LTA alleles showed significantly higher mean scores of the personality traits, impulsivity (*p* = 0.026), EUE (*p* = 0.002), and FF (*p* = 0.022) than boys with obesity having HTA alleles (Supplementary Table S4). Noticeably, boys with normal weight did not show significant differences in mean scores of any personal trait nor any eating behavior (*p* > 0.05), neither girls with normal weight nor girls with obesity for *MAO-A* 30 bp u-VNTR (Supplementary Tables S4, S5).

### 3.4 Multivariate binary logistic regression

The multivariate binary logistic regression model included those variables that showed significant differences during the analysis, namely, sex, age, HIP and LIP, impulsivity, MA, and *MAO-A* 30 bp u-VNTR transcriptional activity. The *FTO*-rs9939609 was also included based on its reported clinical relevance (Table 4). The stratified multivariate model suggested that boys having LTA alleles exhibited four times higher odds of being obese compared with HTA of *MAO-A* 30 bp u-VNTR (OR = 4.44; 95% CI = 1.18–16.63; *p* = 0.027).

**TABLE 4:** Multivariate binary logistic regression for obesity status stratified by sex.

Variable	Boys (*N* = 63)			Girls (*N* = 71)		
	Adjusted OR^b^	95% CI	*p*-value	Adjusted OR^b^	95% CI	*p*-value
Age^a^ ≤9 >9	Reference 0.62	--- 0.17–2.29	--- 0.47	Reference 2	--- 0.56–7.15	--- 0.28
*FTO-*rs9939609 T/T T/A+ A/A	Reference 0.6	--- 0.12–2.96	--- 0.53	Reference 1.01	--- 0.21–5	--- 0.99
*MAO-A* u-VNTR HTA LTA	Reference **4.44**	--- **1.18–16.63**	--- **0.027**	Reference 1.67	--- 0.51–5.47	--- 0.39
Personality traits						
High intensity pleasure^a^ ≤13 >13	Reference 0.84	--- 0.23–3.14	--- 0.8	Reference 0.4	--- 0.11–1.44	--- 0.16
Low intensity pleasure^a^ ≤7 >7	Reference 2.2	--- 0.6–8	--- 0.23	Reference 0.99	--- 0.27–3.57	--- 0.99
Impulsivity^a^ ≤17 >17	Reference 1.28	--- 0.32–5.15	--- 0.73	Reference 1.64	--- 0.4–6.82	--- 0.49
Eating behavior						
Moderate appetite^a^ ≤11 >11	Reference 1.51	--- 0.35–6.46	--- 0.58	Reference 0.6	--- 0.16–2.27	--- 0.45

HTA, high transcriptional activity; LTA, low transcriptional activity. Significant associations are in bolds.

^a^ Adjustment included all of the variables listed in the Table.

^b^ The cut-points were calculated with the median of the obtained score.

## 4 Discussion

The present study evaluated the association of the genetic variants *FTO-*rs9939609 and *MAO-A* 30 bp u-VNTR, along with eating behavior and personality traits with obesity in Mayan children from Mexico. We found significantly higher mean scores in the personality trait HIP and in the eating behavior MA in normal-weight children vs. obese. Heterozygous children T/A of *FTO*-rs9939609 exhibited significantly lower LIP (personality trait) and MA (eating behavior) scores than children with wild-type *FTO*. Additionally, only boys with LTA alleles of *MAO-A* 30 bp u-VNTR showed higher mean scores for the personality traits of impulsivity and anxiety. Moreover, only obese boys with LTA alleles showed significantly higher mean scores of impulsivity, as well as for the eating behaviors of EUE and FF. However, in multivariate analysis, only the LTA alleles of the *MAO-A* u-VNTR were associated with obesity, particularly in boys of this Mayan children population.

By 2015, the accumulated evidence gave birth to the behavioral susceptibility theory (BST), which proposes that “obesity-related genes” influence weight gain partly through their effects on appetite and that variations in appetite emerge in the latter stages of life (Llewellyn and Wardle, 2015). The behavioral component of the BST is evaluated through the CEBQ. This instrument assesses food approach traits [VEI (FR and EOE), EF, and DD] and food avoidance traits [MA (SR and SE), FF and EUE) in our local validation of the instrument. In this respect, some of the CEBQ traits have shown to be positively (EF and FR) or negatively (SR and FF) associated with BMI or BMI z-scores (WHO or CDC) (Sánchez et al., 2016; Boswell et al., 2018; Pesch et al., 2018). Such information has also been synthesized in a recent comprehensive meta-analysis that found higher CEBQ food approach trait scores and lower food-avoidant trait scores in obese children (Kininmonth et al., 2021).

Conversely, a few reports show no association between obesity (measured as BMI) and eating behavior (Hayes et al., 2016; Lora et al., 2016). Our results partially agree with these findings since one of the six CEBQ dimensions differed between obese and normal-weight children, with MA showing higher scores in children with normal weight. As expected, parents of children with normal weights describe them as having more moderate eating habits, which might contribute to their healthy weight maintenance.

Otherwise, independently of BMI status, the MA mean score was higher in wildtype T/T children than T/A heterozygous for *FTO*-rs9939609, suggesting the MA as an eating behavior consistent with adequate satiety in children with wildtype *FTO*. The MA score is lower in those carrying one *FTO* risk allele, as in the T/A genotype, implying a lower threshold for satiety in heterozygous children (according to the parent’s testimony). Taking into account that MA in our validated CEBQ questionnaire estimates SR, other studies, such as that of Obregon-Rivas et al., 2018 (Obregón Rivas et al., 2018), demonstrated association of the *FTO*-rs9939609 gene with eating behavior traits, dependent of sex and nutritional status in Chilean children: normal weight girls carriers of the A allele showed higher SR and SE scores; normal weight boy carriers of the A allele showed higher scores on the negative affect subscale; overweight boys with the A allele showed higher FR, EOE, and lower SR and SE. In contrast, in preschool children from the Generation R study, it was found that the minor allele A leads to increased food responsiveness and better emotional control (Velders et al., 2012).

In adults, the *FTO*-rs9939609 has been associated with poorer eating behavior in an age-dependent fashion. For example, it has been reported a higher decline of emotional eating with age in the A/A+ T/A genotype group compared to the T/T group (Abdella et al., 2019). The *FTO* gene variation (i.e., rs9939609 A/A+ T/A vs. T/T) has been associated with increased dietary intake from unhealthy eating behaviors such as sweet, fat, and carbohydrate cravings from fast food (Abdella et al., 2019). Also, *FTO* A allele has also been associated with increased severity of eating disorders like binge eating behavior or psychopathological features, including emotional eating and disorder of corporeality (Castellini et al., 2017). In Mexican young adults (20.14 ± 3.95 years) from Guadalajara, Mexico, the *FTO*-rs9939609 genotype T/T was associated only with higher EUE in bivariate models (OR: 1.8; 95% CI: 1.1–9.1; *p* = 0.014) (Rivera-Iñiguez et al., 2024). In the same Mexican State, volunteers aged 18–25 years carrying the *FTO*-rs9939609T/A + A/A genotypes showed a higher consumption of products with added sugars than those participants with the T/T genotype (Madrigal-Juarez et al., 2023). In the context of metabolic syndrome components, a study in women from Mayan communities of Chiapas, Mexico, found an association of hyperglycemia with *FTO*-rs9939609 in the dominant model (T/T vs. T/A + A/A; OR_adjusted_: 2.6; 95% CI: 1.3–5.3; *p =* 0.007) (Núñez-Ortega et al., 2021).

The effect of *FTO* genotypes on psychological aspects might be modified by vitamin D intake; insufficient vitamin D intake might be an additional contributor to the effect of *FTO* genotypes on eating behaviors and mental health (Mehrdad et al., 2022). One pathway of the *FTO* effect on adiposity is by influencing dopamine signaling, given that *FTO* variants associated with obesity are also associated with several behaviors or disorders dependent on dopamine. For example, *FTO* knockout in mice has been shown to impair control of dopamine receptors D2 of neuronal activation and DA-dependent-regulation of locomotor activity and reward sensitivity (Sun et al., 2017). A neuro-image study showed that carriers of *FTO* variants have reduced longitudinal functioning in the medial prefrontal cortex, consistent with higher rates of impulsivity, behavioral disinhibition, and risky decision-making (Chuang et al., 2015). In this study, LIP (personality trait) differed according to *FTO*-rs9939609 genotypes, observing higher scores in T/T than in heterozygous T/A.

The abovementioned is striking since the higher LIP score suggests high sensitivity to the enjoyment of situations related to low stimuli, complexity, and novelty, suggesting that children with the A allele with lower LIP scores need more stimuli to get the optimal satiety threshold. Prediction analysis *in silico* has suggested that *FTO* risk variants could directly affect modulating binding sites of transcription factors as a regulatory function during brain development (Saucedo-Uribe et al., 2019). Even so, it is suggested that *FTO* in the basal ganglia regulates a circuit that steers movements and regulates reactions to novelty, exploration, motor function, and timing behavior *via* D2R medium spiny neurons. However, these changes do not predispose to weight gain or altered food reward in mice (Ruud et al., 2019). The latter might explain our finding on the *FTO* contribution independently of BMI status.

Notably, the *FTO-*rs9939609 was not associated (in uni- and multivariate models) with obesity in the studied children. A meta-analysis found 37% higher odds of being obese children in the group of the allele of risk A compared with the wild type T (OR = 1.37; 95% CI = 1.25–1.51; *p* ≤ 0.001). Such a result was still significative after stratifying by Caucasian (OR = 1.37; 95% CI = 1.18–1.6; *p* ≤ 0.001) or Asian (OR = 1.34; 95% CI = 1.25–1.43; *p* ≤ 0.001) ethnicity (Dastgheib et al., 2021). However, the association did not remain in the mixed population group (OR = 1.23; 95% CI = 0.77–1.95; *p* = 0.38). The latter was observed for Brazilian and Chilean children, two Latin-American populations that share a genetic admixture background with Mexico. Some studies in the Mexican population also found no association of BMI or other indicators of obesity [i.e., waist circumference, body fat (%), and energy intake] with the risk allele A from *FTO-*rs9939609 (Villalobos-Comparán et al., 2017; Chama-Avilés et al., 2023). In Yucatan, Mexico, our group previously reported the univariate association of obesity with the presence of the heterozygous AT and the risk allele A only in girls (González-Herrera et al., 2019b). The lack of association in the present sample of children might be due to limitations on sample size, given that none of the obese children carried the A/A genotype, whereas 5% of normal-weight cases did. However, it has been demonstrated that the association between *FTO* variation and obesity is sex-dependent. For example, the rs1421085 and rs9939609 affect genetic susceptibility for obesity only in girls, whereas rs8057044 and CNV are associated with overweight status only in boys. Furthermore, the SNP rs1421085 of the *FTO* gene was strongly associated with obesity in Mayan children instead of the rs9939609 (González-Herrera et al., 2019b).

A careful inspection of the evidence related to the BST allows the identification of the genetic predisposition as an unmodifiable factor for obesity. In this way, some polymorphisms in genes involved in the dopaminergic pathway, such as the 30 bp u-VNTR of the *MAO-A*, have shown consistent associations. The *MAO-A* is found on chromosome Xp11.3, thus generating heterozygous or homozygous females and hemizygous males. Further research showed that 3R and 4R alleles conferred low (LTA) and high (HTA) transcriptional activity, respectively. A report on alcoholic males showed significantly higher BMI in those cases with LTA than in those with HTA (Ducci et al., 2006). Also, a study on Chinese adolescents found that males with obesity had 1.85 times the odds of being in the LTA group compared with those in the HTA (OR = 1.85; 95% CI = 1.18–2.94; *p* = 0008) (Fuemmeler et al., 2008). Our findings agree with these previous reports since boys with LTA alleles exhibited four times the odds of being obese than boys with HTA alleles, suggesting the role of the dopaminergic pathway through the *MAO-A* gene in obesity pathogenesis, particularly in hemizygous boys. Human MAO-A is strongly expressed in adipocytes, where the non-neuronal transporter SLC22A3 allows the transportation and degradation of catecholamines. It has also been suggested that adipocytes are involved in establishing adrenergic tone and controlling thermogenic activity in humans since inhibition of MAOA can induce human adipocyte browning *in vivo* (Solivan-Rivera et al., 2022). MAO-A is the predominant isoform in human abdominal adipose and vascular tissues; it is overexpressed during inflammation and contributes to endothelial dysfunction (Sturza et al., 2019).

In the present study, we found significant differences in some personality traits, namely, the HIP and LIP, and impulsivity according to BMI, *FTO-*rs9939609, and *MAO-A* 30 bp u-VNTR. However, in multivariate logistic regression models, neither the *FTO-*rs9939609 nor the personality traits were associated with obesity. In this regard, two reports in large Dutch cohorts of children found no linear association between BMI z-scores and impulsivity (from TMCQ) among non- and overweight participants (Scholten et al., 2014; Sleddens et al., 2016). As a qualitative contribution of the present study, we observed that girls’ parents described their daughters as “audacious” and active seekers of intense, long-lasting, incongruent, and novel sensations related to pleasure or enjoyment. A possible explanation is that all girls look for stimulation, although normal-weight ones look outside their bodies. In contrast, obese ones look inside their bodies, using food as a vehicle for such stimuli.

On the other hand, we found significantly higher impulsivity scores in boys with *MAO-A* 30 bp u-VNTR LTA than those in the HTA group. Although it might be hypothesized that obese boys are impulsive and eat by anxiety rather than appetite, none of these “pre-conceived” ideas were corroborated in multivariate models. This finding agrees with previous reports in which boys with higher neuroticism (where anxiety and impulsivity are contained) showed higher consumption of sweetened beverages and low consumption of fruits and vegetables (Vollrath et al., 2012). It also might be hypothesized that parent beliefs regarding the “natural impulsivity” of boys promote weight gain unconsciously. Finally, we cannot discard additional unexplored factors mediating between the *MAO-A* 30 bp u-VNTR LTA and obesity. For example, a recent study found a hindbrain dopaminergic circuit in the caudal ventral tegmental area innervating DRD1-expressing neurons within the lateral parabrachial nucleus that regulates satiety and meal structure (Han et al., 2021). Therefore, mutations in such a critical circuit might explain the phenotype related to weight excess. The *MAO-A* 30 pb VNTR associated with LTA might operate differently in boys compared to girls, or there is also the possibility of different upbringings for boys and girls. This upbringing would consist of what food refers to and what is taught and allowed. In men, there may be a tendency toward greater indulgence and care. Conversely, women might receive better nutritional education, as excess weight and obesity are less tolerated in them compared to men. However, further studies and evidence are necessary to fully understand these differences.

To our knowledge, the present study is the first to evaluate the association of the genetic variants of *FTO* and *MAO-A*, along with eating behavior and personality traits with childhood obesity in Mexico. Also, some residual confusion generated by overweight children was ruled out during the design stage. Nevertheless, some limitations must be acknowledged. Firstly, the psychological tests employed to evaluate personality traits and eating behavior in this sample of children might be influenced by parental perceptions, including gender stereotypes or the inadvertent normalization of certain behaviors. Secondly, the sample size might have influenced the low prevalence of the A/A genotype of *FTO*-rs9939609 and the sex-stratified analyses. Thirdly, although the nutritional perspective was indirectly evaluated through the evaluated tests, a more integrative approach should evaluate at least 24-h dietary records. Finally, the study’s observational nature makes it impossible to draw causality-related conclusions, although some associations persisted even in multivariate models. Despite these limitations, and given the instrument’s inherent limitations related to parent perception, future analyses in a larger sample are required to incorporate eating behavior as an explicative obesity variable.

## 5 Conclusion

In the present study, some dimensions of the personality traits and eating behavior tests differed by sex, nutritional status, *FTO*-rs9939609, and *MAO-A* 30 bp u-VNTR. However, in multivariate analysis, only the latter retained its association with obesity in boys. Childhood obesity still represents a complex phenomenon that should be evaluated, at least from the psychological, genetic, nutritional, sex, and sociocultural perspectives. Moreover, the findings support that the factors associated with childhood obesity in a determined population might not necessarily be explicative in others.

## Data Availability

The raw data supporting the conclusions of this article will be made available by the authors, without undue reservation.
