# Supplementary material for: Association between personality traits, eating behaviors, and the genetic polymorphisms FTO-rs9939609 and MAO-A 30 bp u-VNTR with obesity in Mexican Mayan children
Source: Front Genet. 2024 Jul 26;15:1421870. doi: 10.3389/fgene.2024.1421870 (PMC11310059; doi:10.3389/fgene.2024.1421870)
Supplement: Supplementary file 1 [file Table1.docx]

**Association between personality traits, eating behaviors, and the genetic polymorphisms *FTO*-rs9939609 and *MAO-A* 30 bp u-VNTR with obesity in Mexican Mayan children**

Luis Alberto Vázquez-Pérez^1^, Mónica Hattori-Hara^2^, Gloria Arankowsky-Sandoval^1^, Gerardo Pérez-Mendoza^1^, Rodrigo Rubi-Castellanos^1^, Jorge Aarón Rangel-Méndez^1^, Doris Pinto-Escalante^1^, Thelma Canto-Cetina^1^, Lizbeth González-Herrera^1^*

^1^Centro de Investigaciones Regionales “Dr. Hideyo Noguchi”, Universidad Autonoma de Yucatan. C.P. 97225 Merida, Yucatan, Mexico.

^2^Secretaría de Educación del Gobierno del Esatdo de Yucatán (SEGEY)

^*^Corresponding author: Dr. Lizbeth Gonzalez-Herrera. Laboratorio de Genética. Centro de Investigaciones Regionales. Edificio Inalámbrica. Universidad Autonoma de Yucatan. Calle 43 s/n x 96. Fraccionamiento Paseo de las Fuentes. Mérida, Yucatán, Mexico, C.P. 97225. Telephone: +52 999 9245755 Ext. 1240 Fax: 52 999 9236120. E-mail: [lizbeth@correo.uady.mx](mailto:lizbeth@correo.uady.mx).

**Table S1.** Genotypic and allelic frequencies of *FTO*-rs9939609 and *MAO-A* u-VNTR

| **Sex** | ***FTO-*rs9939609^a^** | | | | | **Sex** | ***MAO-A* u-VNTR^a, b^** | | | | |
| --- | --- | --- | --- | --- | --- | --- | --- | --- | --- | --- | --- |
|  | **T/T** | **T/A** | **A/A** | **T** | **A** |  | **3R/3R** | **3R/4R** | **4R/4R** | **3R** | **4R** |
| Boys  (*N=* 88, 2*N*= 176) | 68  (0.77) | 18  (0.21) | 2  (0.02) | 154  (0.87) | 22  (0.13) | Boys  (*N=* 63) | N/A | N/A | N/A | 38  (0.60) | 25  (0.40) |
|  | HWE: 0.30 | | | | |  | HWE: N/A | | | | |
| Girls  (*N=* 98, 2*N*= 196) | 80  (0.82) | 14  (0.14) | 4  (0.04) | 174  (0.89) | 22  (0.11) | Girls  (*N=* 71, 2*N*= 142) | 13  (0.18) | 30  (0.42) | 28  (0.40) | 56  (0.39) | 86  (0.61) |
|  | HWE_1_: **0.001** HWE_2_: 1.00 | | | | |  | HWE: 0.32 | | | | |
| Total  (*N=* 186, 2*N*= 372) | 148  (0.80) | 32  (0.17) | 6  (0.03) | 328 (0.88) | 44  (0.12) | Total  (*N=* 134, 2*N=* 205) | N/A | N/A | N/A | 94  (0.46) | 111  (0.54) |
|  | HWE_1_: **0.04** HWE_2_: 0.93 | | | | |  | HWE: N/A | | | | |

HWE: Hardy-Weinberg Equilibrium; HWE_1_: χ^2^ test; HWE_2_: χ^2^ test for trend (Armitage trend test); N/A: not apply.

^a^Data is presented as *N* (frequency)

^b^Boys are hemizygous for *MAO-A* u-VNTR

**Table S2.** Personality traits and eating behavior scores according to *FTO* genotypes

|  | **T/T**  ***N=* 148** | **T/A**  ***N*= 32** | **A/A**  ***N=* 6** | **T/T versus T/A**  *p*-value^a^ | **T/T vs. A/A**  *p-*value^a^ |
| --- | --- | --- | --- | --- | --- |
| Personality traits, mean±SD  Anxiety  Impulsivity  High intensity pleasure  Low intensity pleasure | 15.71±4.27  18.04±5.36  13.10±6.55  7.39±4.82 | 16.03±4.22  17.57±5.38  12.83±5.42  5.12±3.66 | 12.33±4.36  15.10±4.15  16.00±6.54  9.16±2.71 | 0.698  0.653  0.805  **0.002** | 0.063  0.093  0.287  0.132 |
| Eating behavior, mean±SD  Voracity and emotional intake  Moderate appetite  Enjoyment of food  Desire to drink  Emotional undereating  Food fussiness | 12.80±7.68  12.43±6.02  12.66±3.83  5.11±3.27  3.86±2.98  7.41±3.40 | 13.59±8.48  10.29±4.7  13.28±4.02  5.28±3.83  3.67±2.67  7.81±4.21 | 14.50±6.71  13.50±3.03  13.00±2.36  4.33±3.01  4.00±2.75  5.33±3.07 | 0.627  **0.027**  0.425  0.815  0.721  0.615 | 0.545  0.421  0.737  0.535  0.903  0.105 |

^a^Student’s t test. Significant *p*-values are in bolds.

**Table S3.** Personality traits and eating behavior scores in boys and girls according to *MAO-A* u-VNTR

transcriptional activity

| **Population** | **Psychological profile** | **Transcriptional activity** | | ***p-*value*^a^*** |
| --- | --- | --- | --- | --- |
|  |  | **LTA** | **HTA** |  |
| **Total** | Personality traits, mean±SD  Anxiety  Impulsivity  High intensity pleasure  Low intensity pleasure | ***N*= 51**  15.86±4.96  18.76±5.50  12.58±6.65  7.20±4.96 | ***N*= 83**  15.28±3.19  16.73±4.54  13.85±5.98  7.09±4.51 | 0.455  **0.027**  0.267  0.897 |
|  | Eating behavior, mean±SD  Voracity and emotional intake  Moderate appetite  Enjoyment of food  Desire to drink  Emotional undereating  Food fussiness | 13.45±7.90  12.26±5.51  12.65±3.55  4.92±3.56  4.12±3.23  7.62±3.82 | 12.52±7.29  11.80±6.10  12.82±3.98  5.71±2.96  3.43±2.74  7.35±2.91 | 0.497  0.653  0.797  0.187  0.207  0.665 |
| **Girls** | Personality traits, mean±SD  Anxiety  Impulsivity  High intensity pleasure  Low intensity pleasure | ***N*= 13**  15.85±4.53  17.75±4.75  13.29±6.78  7.47±4.76 | ***N*= 58**  15.23±3.27  16.96±4.90  13.35±5.87  7.17±4.25 | 0.647  0.596  0.976  0.836 |
|  | Eating behavior, mean±SD  Voracity and emotional intake  Moderate appetite  Enjoyment of food  Desire to drink  Emotional undereating  Food fussiness | 12.11±7.15  12.62±5.93  12.12±4.0  4.16±3.44  4.02±3.23  6.54±3.29 | 13.61±6.91  11.56±3.53  12.83±3.87  5.67±2.34  3.85±2.36  6.96±3.03 | 0.500  0.544  0.567  0.131  0.860  0.678 |
| **Boys** | Personality traits, mean±SD  Anxiety  Impulsivity  High intensity pleasure  Low intensity pleasure | ***N*= 38**  18.87±5.47  19.90±6.11  11.78±6.51  6.90±5.23 | ***N*= 25**  15.33±3.16  16.48±4.18  14.41±6.18  7.00±4.88 | **0.001**  **0.008**  0.105  0.938 |
|  | Eating behavior, mean±SD  Voracity and emotional intake  Moderate appetite  Enjoyment of food  Desire to drink  Emotional undereating  Food fussiness | 14.96±8.51  11.85±5.04  13.24±2.90  5.78±3.55  4.23±3.27  8.84±4.05 | 11.30±7.66  12.08±8.15  12.80±4.17  5.75±3.59  2.96±3.10  7.80±2.76 | 0.075  0.900  0.648  0.974  0.119  0.230 |

HTA: high transcriptional activity; LTA: low transcriptional activity

**^a^**Student’s t test. Significant *p*-values are in bolds.

**Table S4.** Personality traits and eating behavior scores in boys and girls with obesity according to

*MAO-A* 30 bp u-VNTR transcriptional activity

| **Population** | **Psychological profile** | **Transcriptional activity** | | ***p-*value^a^** |
| --- | --- | --- | --- | --- |
|  |  | **LTA** | **HTA** |  |
| **Girls** | Personality traits, mean±SD  Anxiety  Impulsivity  High intensity pleasure  Low intensity pleasure | *N*= 5  15.32±3.64  17.45±3.99  10.26±6.73  6.50±4.63 | *N*= 16  17.00±3.34  15.83±4.13  12.67±8.82  7.17±2.79 | 0.358  0.432  0.518  0.759 |
|  | Eating behavior, mean±SD  Voracity and emotional intake  Moderate appetite  Enjoyment of food  Desire to drink  Emotional undereating  Food fussiness | 11.71±6.41  11.04±4.65  12.18±3.63  3.71±3.77  4.85±3.00  7.67±2.94 | 13.69±4.99  12.33±2.59  12.70±3.55  4.00±2.28  2.67±2.65  4.85±3.00 | 0.526  0.553  0.778  0.870  0.145  0.062 |
| **Boys** | Personality traits, mean±SD  Anxiety  Impulsivity  High intensity pleasure  Low intensity pleasure | *N*= 24  15.97±5.70  21.01±6.82  11.20±6.67  7.63±6.08 | *N*= 6  14.77±1.68  16.00±4.33  15.20±5.16  8.33±5.12 | 0.374  **0.026**  0.110  0.773 |
|  | Eating behavior, mean±SD  Voracity and emotional intake  Moderate appetite  Enjoyment of food  Desire to drink  Emotional undereating  Food fussiness | 14.46±8.15  11.27±4.03  13.59±2.90  5.50±4.13  3.70±2.79  8.67±4.20 | 13.6±12.48  12.17±12.10  13.50±6.56  5.50±4.63  1.00±1.54  6.50±1.00 | 0.872  0.857  0.973  1.000  **0.002**  **0.022** |

LTA: low transcriptional activity; HTA: high transcriptional activity.

^a^Student’s t test. Significant *p*-values are in bold.

**Table S5.** Personality traits and eating behavior scores in boys and girls with normal weight according to

*MAO-A* 30 bp u-VNTR transcriptional activity

| **Population** | **Psychological profile** | **Transcriptional activity** | | ***p*-value^a^** |
| --- | --- | --- | --- | --- |
|  |  | **LTA** | **HTA** |  |
| **Girls** | Personality traits, mean±SD  Anxiety  Impulsivity  High intensity pleasure  Low intensity pleasure | *N*= 8  15.68±4.47  17.94±5.23  14.82±6.52  7.75±4.82 | *N*= 42  14.76±3.17  17.27±5.13  13.55±5.05  7.18±4.62 | 0.578  0.739  0.601  0.757 |
|  | Eating behavior, mean±SD  Voracity and emotional intake  Moderate appetite  Enjoyment of food  Desire to drink  Emotional undereating  Food fussiness | 12.32±7.73  13.29±6.49  12.14±4.30  4.22±3.25  3.61±3.37  6.93±3.81 | 13.59±7.44  11.35±3.78  12.87±4.04  6.13±2.19  4.18±2.23  6.77±3.10 | 0.668  0.412  0.656  0.110  0.645  0.910 |
| **Boys** | Personality traits, mean±SD  Anxiety  Impulsivity  High intensity pleasure  Low intensity pleasure | *N*= 14  16.07±5.29  18.30±4.23  13.30±6.26  5.84±3.15 | *N*= 19  15.21±3.36  16.67±4.36  14.17±6.78  6.45±4.98 | 0.593  0.280  0.703  0.667 |
|  | Eating behavior, mean±SD  Voracity and emotional intake  Moderate appetite  Enjoyment of food  Desire to drink  Emotional undereating  Food fussiness | 15.88±9.73  13.07±6.69  12.70±3.00  6.53±2.18  5.15±4.07  9.30±4.04 | 10.39±5.82  12.00±7.12  12.45±3.37  6.00±3.37  3.72±3.26  7.94±2.88 | 0.060  0.658  0.822  0.584  0.278  0.282 |

LTA: low transcriptional activity; HTA: high transcriptional activity.

^a^Student’s t test. Significant *p*-values are in bold.
